# United States Veterinary Medical Association

**Published:** 1893-02

**Authors:** 


### SOCIETY PROCEEDINGS.

### UNITED STATES VETERINARY MEDICAL ASSOCIATION.

### MEETING OF THE COMITIA MINORA.

A meeting of the Comitia Minora of the United States Veterinary Medical Association was held at the Arena, New York City, on January 20th. The meeting was called to order at 10 p. m., Chairman Huidekoper presiding. On roll call Williams, Liautard, Huidekoper, Clement, Robertson, Hoskins, Turner and Winchester were present. Absent—Hollingsworth and Stewart. The Secretary read letters and telegrams of regret of their inability to be present from Salmon and Baker of the International Committee, and Hollingsworth and Stewart of the Comitia Minora.

A resolution, submitted at a former meeting of the Comitia Minora, empowering the President to secure the title and a synopsis of the essays and papers to be submitted at the International Congress thirty days before said meeting, in order to furnish to such members of the Association as he may select, that they may prepare themselves to discuss said papers at our meeting, was, after some discussion, adopted with but one dissenting voice.

The Secretary reported his correspondence with Mr. Bonney, manager of the World's Fair Congresses, relative to our International Meeting being one of the series of Congresses being planned to be held during the World's Fair within the grounds, was then taken up and discussed. The time of meeting, owing to a lack of information, the matter was finally submitted, under a resolution, to a sub-committee of three to be appointed by the President. Said committee to have power to determine the time, of meeting and to report in six weeks. The President announced the following committee: Withers, Hoskins and Huidekoper.

Chairman Hoskins, of the Publication Committee, reported an estimate on it offered for publication of the same, in accordance with a resolution passed at the Boston meeting, which was indorsed by the Comitia Minora, and the Publication Committee was directed to proceed with its completion.

A communication as follows was received from the President of the New York Veterinary Medical Society:

*The Officers and Comitia Minora of the U. S. V. M. A.:*

*Dear Sirs*:—We beg to inform you that the officers and Committee of Arrangements of the New York State Veterinary Medical Society have been appointed a committee, and have been empowered to act in conjunction with your committee in regard to arranging our summer meeting so as to aid in the general plan for reception of visitors at the time of the International Congress.

Very respectfully,

R. S. Huidekoper,

*President N. Y. State Vet. Med. Society.*

January 20th, 1893.

The following report of the International Committee was then submitted and after some consideration the report was received and adopted :

REPORT OF THE INTERNATIONAL COMMITTEE OF THE UNITED STATES VETERINARY MEDICAL ASSOCIATION.

We beg to report the following outline, or proposition, of what should be done for combining an International Veterinary Congress with the 30th Annual Meeting of the United States Veterinary Medical Association, to be held at Chicago in 1893:

I. To issue an invitation to the official veterinarians of foreign Governments, directors of Veterinary Colleges, presidents of representative Veterinary Societies, and to individual celebrated veterinarians of Great Britain, Europe and other countries, as follows:

*Respected Sir and Colleague* :—The United States Veterinary Medical Association and first International Veterinary Congress of America, beg to announce its 30th Annual Meeting to be held at Chicago on..... ............. ........, 1893. You and representatives of your.........................are respectfully invited to attend.

The subjects to be discussed will be: "Veterinary Education;" "Tuberculosis;" "Animal Food."

The reporters upon these subjects will be announced in a later bulletin.

It is hoped that our foreign colleagues will make the occasion of the World's Fair, at Chicago, the time for a visit to America, and that they will assist at the International Veterinary Congress.

We beg that the delegates from European countries will arrange to arrive in New York about....... .................and accept the following invitation:

...... ... ........ A reception by the Veterinarians of New York City.

..................... A reception by the Pennsylvania State Veterinary Medical Association in Philadelphia.

.. ................. A reception by the Maryland Veterinary Society with a visit to Washington, the capital of the country.

..................... A reception by the New York State Veterinary Medical Society at Niagara Falls.

..................... A reception by the United States Veterinary Medical Association at Chicago.

All veterinarians are invited to become subscribing members of the 30th Annual Meeting of the United States Veterinary Medical Association and First International Veterinary Congress of America. The fee is two dollars ($2.00), which entitles the subscriber to a seat at the meetings in Chicago and to a copy of the proceedings, which will be published promptly after the Congress.

Acceptance of the above invitation and subscriptions for subscribing membership will be sent to Dr. J. L. Robertson, Treasurer, 409 Ninth Ave., New York City.

II. The committee recommends that immediate steps be taken to obtain the harmonious action of local and State societies in aiding in the reception of foreign visitors.

III. We recommend that we be given power to act in regard to all details, subject to the approval of the president of the Association.

For the Committee,

RUSH S. HUIDEKOPER, *Chairman.*

The Committee on Honorary Membership submitted a synopsis of the progress of their work, which was approved so far as submitted.

The Committee on Veterinary Education, through Chairman Liautard, desired an expression of information as to the scope and topics, etc., to be embraced under the subject which said Committee was to plan for consideration at our Congress After considerable discussion, it was determined that said Committee would continue on the plan which they proposed of confining their work, principally, under the heading of Schools and Colleges. A report of Progress submitted by the Committee on Veterinary Education was approved.

On motion of Dr. Peters, seconded by a member of the profession present, a vote of thanks was accorded to the New York Veterinary Surgeons for the hospitable manner in which they had entertained the members of the various Committees who were present from outside of the city, which was unanimously adopted.

The Committee on Tuberculosis, through Chairman Clement, submitted their report, which was received and their plan indorsed.

A motion was then offered that a Committee of three be appointed to act in conjunction with the Illinois, Indiana and Iowa local Committees to complete the local arrangements for the meeting at Chicago. Said motion was adopted, and the President announced that he would make his appointment at a later date.

Dr. Clement brought up the subject of the need of interpreters for our Congress, which was referred to the International Committee, with directions that they provide the same for our meeting.

Chairman Lyman, of the Prize Committee, desired information as to the duties of said Committee, which, after some discussion, ended in the President directing the Secretary to place in the hands of the Chairman a report of all the resolutions, etc., relative to the duties of said committee, that appeared in the by-laws and amendments of the Association. Editors Huidekoper of the JOURNAL OF COMPARATIVE MEDICINE, and Liautard of the *American Veterinary Review*, offer, in addition to the money prize of the Association for the best paper, silver plate to the value of $50.00 each, for a prize paper submitted to the meeting at Chicago, under the conditions and rules, governing the same, of our Association.

Dr. Lyman brought up the subject of incorporation of our Association, or the making of our Association an incorporated body. After some discussion a motion was made that the committee appoint a committee of three to consider the advisability of this movement. Said committee to report at our next meeting, which was carried; after which a motion to adjourn prevailed.

Dr. W. L. WILLIAMS, *President.*

Dr. W. HORACE HOSKINS, *Secretary.*

The following committees reported :

The International Committee meeting, January 20, 1893. Members present—Huidekoper, Liautard, Williams, Clement, Hoskins, Robertson. Absent—Salmon, Baker, Schwartzkopff and Stickney. Chairman Huidekoper offered a report incorporating a plan for the meeting and an outline of the proposed invitation to foreign delegates, which were, on motion, adopted; after which the committee adjourned.

The Committee on Honorary Membership. Members present—Liautard, Huidekoper and Pearson. A list, comprising a quota of three of the leading recognized members of the profession in the different countries of the world, was submitted, and so far as completed was approved.

The Committee on Tuberculosis. Members present—Clement, Pearson and Peters. After some discussion the following report was adopted for submission to the Comitia Minora :

It is proposed to have Dr. Peters treat the subject from a clinical and sanitary standpoint, with the carrying on of feeding and inoculation experiments.

Dr. Pearson to treat the subject from a laboratory standpoint, with special references to the use of tuberculin as a means of diagnosis and cure. The inoculation of other ptomaines for comparison of results, and the carrying on of feeding experiments.

The Chairman to treat of the subject from a general standpoint, and to incorporate the reports of the other members verbatim.

A. W. CLEMENT, *Chairman*,
AUSTIN PETERS,
LEONARD PEARSON.

